# Mechanical Properties of Composites Used in High-Voltage Applications

**DOI:** 10.3390/polym8070260

**Published:** 2016-07-14

**Authors:** Andreas Moser, Michael Feuchter

**Affiliations:** 1 Institute of Material Science and Testing of Polymers, Montanuniversitaet Leoben, 8700 Leoben, Austria; 2 Polymer Competence Center Leoben, 8700 Leoben, Austria; michael.feuchter@pccl.at

**Keywords:** dynamic mechanical analysis, tensile, bending, impact behavior, thermosets

## Abstract

Materials used in high voltage applications have to meet a lot of regulations for their safety and functional usage during their lifetime. For high voltage applications the electrical properties are the most relevant designing criteria. However, the mechanical properties of such materials have rarely been considered for application dimensioning over the last decades. This article gives an overview of composite materials used in high voltage applications and some basic mechanical and thermo-mechanical characterization methods of such materials, including a discussion of influences on practically used epoxy based thermosets.

## 1. Introduction

**Figure 1:**
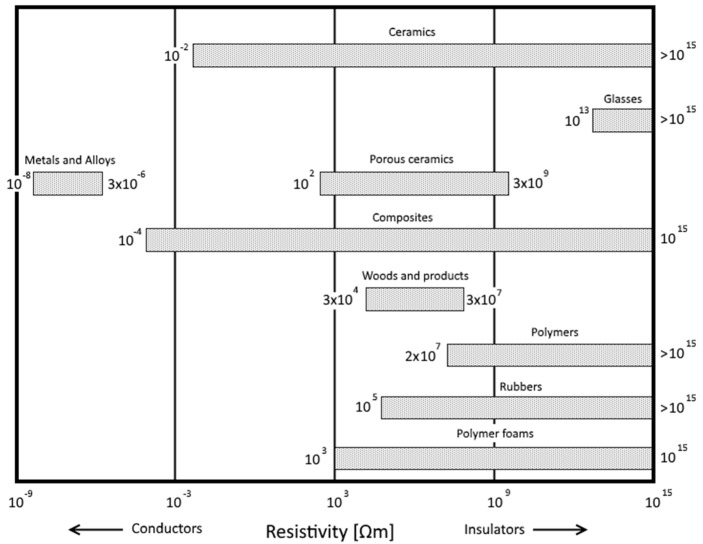
Electrical resistivity chart of various material classes.

For all electrical and electronic applications, the material’s ability to insulate and protect the conductive circuit is of high importance for the safety and functional operation. Solid organic materials used in electrical engineering are paper, wood, wax, leather, as well as a number of natural and synthetic resins, rubbers, and polymers [1] (see electric resistivity chart in Figure 1). Since their introduction in the early 1970s, insulators built of polymer materials have been heavily used as suitable replacements for porcelain and glass insulators.

Thermosets are generally reported as having very good dimensional stability, which makes them usable in sheets and bulk molding compounds for encapsulates, coatings, and insulating applications. The processing of thermosets can be done with high precision to form very stable polymer films. For electrical insulating moldings, such as switch housings, connector shells, high voltage insulators, and equipment casings, thermosets are often chosen for their dimensional stability and mechanical performance when subjected to electrical, thermal and environmental stress over time [2]. In the case of electrical equipment, such as high voltage capacitors and printed circuit boards epoxy matrix materials—based on bisphenol A diglycidyl ether (DGEBA/BADGE)—are known to withstand high dielectric breakdown voltage and high temperatures [3]. However, in the construction process of electrical applications, mechanical stresses are not considered to have severe influences on the lifetime of a product, though it is known in polymer engineering that the slightest stress or defect in polymer materials could lead to a failure of the product. Therefore, mechanical, fracture mechanical, and long-term characterization should be considered for the dimensioning of polymeric materials in electrical and electronic applications. In the following review, a brief overview on methods of mechanical and thermo-mechanical characterizations is given including representative examples found in literature. In addition, short background information of these characterization methods is presented.

## 2. Characterization of Thermo-Mechanical Properties of Thermosets

In the case of application requirements, the required temperature range is described as service temperature. For thermosets, the suitability is described as the glass transition temperature (*T*_g_). If the thermoset is loaded during its lifespan it is of immense importance for the *T*_g_ to be well above the service temperature. Therefore, a good understanding of thermal and thermo-mechanical properties of thermosets is necessary. To characterize the thermo-mechanical behavior of polymeric materials, the use of dynamic mechanical analysis (DMA) provides precise information about the usable service region of a thermoset [1,2,4].

### 2.1. Dynamic Mechanical Analysis

**Figure 2:**
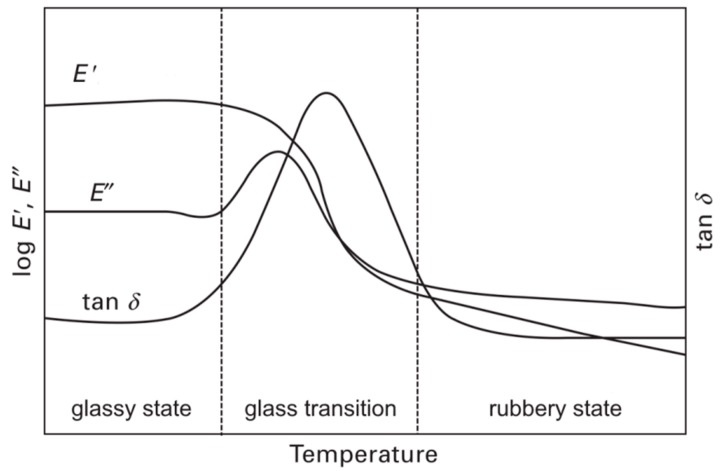
A typical dynamic mechanical analysis (DMA) curve of thermoset polymer.

The DMA is used to characterize the viscoelastic behavior of a polymeric material over a broad range of temperature and frequency. Viscoelastic behavior covers changes in molecular dynamics, which is characterized by localized and cooperative motions in polymers. These motions are responsible for different relaxations processes, which are composed of energy dissipation and mechanical damping. The determination of this viscoelastic behavior is performed via small dynamically applied strain or stress (sinusoidal strain, stress). As a result of the viscoelastic behavior of polymeric materials, a phase lag is introduced between strain and stress. As a result of this dynamic problem, a complex solution is applied and results in a complex modulus: $E^{*}=E^{\prime }+iE^{\prime \prime }$, which describes the mechanical property of the characterized material. This complex modulus breaks down into the storage modulus (*E*′, elastic part) and the loss modulus (*E*″, non-elastic part). The quotient of these values is called the damping factor (tan δ) [5]. The dynamically applied strain or stress can be monitored as a function of frequency and temperature. A typical DMA curve for a thermoset resin is shown in Figure 2. For thermosets this curve can be divided into three characteristic regions. At low temperatures (below the glass transition region) the material is in the glassy state and exhibits a brittle and rigid behavior. In the glass transition region there is a loss in the storage modulus of many decades, and peaks in loss modulus and damping factor appears. The materials’ behavior changes from brittle and rigid to soft and ductile. The width of the damping factor peak may describe the degree of homogeneity and crosslink density in thermoset polymers. Adjacent to the glass transition region is the rubbery state, where the material stiffness is very low and it retains its soft and ductile behavior [6].

### 2.2. Structure Property Relationships in the Glass Transition Region

Through the direct connection of network formation (crosslink density, conversion, etc.) and glass transition (*T*_g_), relationships can be reasonably established on the basis of experimental data. In the case of network formation, mainly two cases (homogenous or inhomogeneous networks) can be distinguished [7].

### 2.3. Homogenous Network

**Figure 3:**
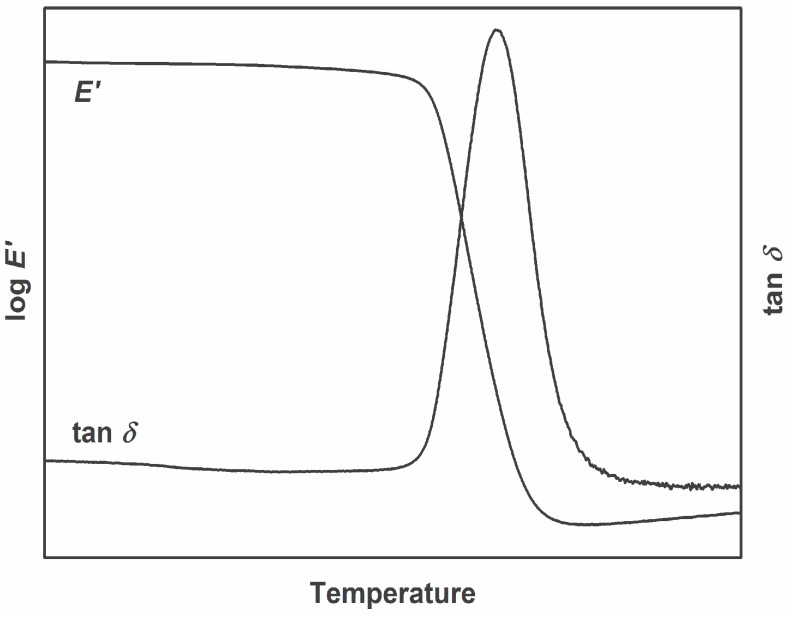
Representative DMA curve of a homogenous thermoset.

Fully cured (homogenous network) can be represented by epoxy-amine systems in terms of stoichiometric mixing. These types of thermosets feature a high conversion rate. Due to the homogenous network, the relaxation process in the glass transition region is consistent. This is represented through an almost spontaneous drop in storage modulus and a sharp and symmetrical peak in damping factor (see Figure 3).

### 2.4. Inhomogeneous Network

**Figure 4:**
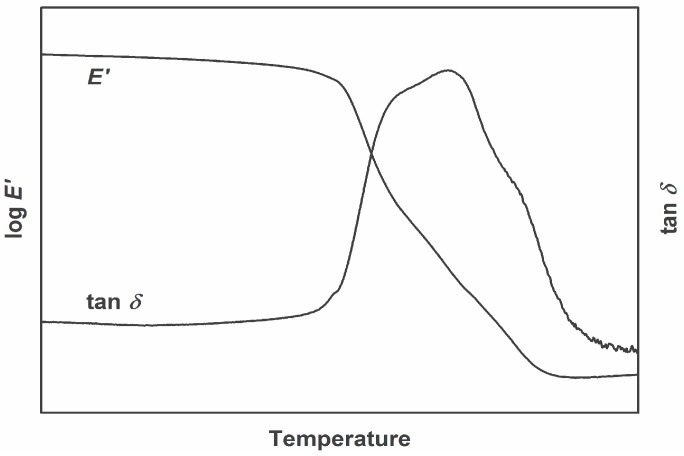
Representative DMA curve of an inhomogeneous thermoset.

As a result of insufficient curing or non-stoichiometric mixture of monomers, the resulting network of thermosets may be inhomogeneous. This leads to regions in the polymeric material, where crosslink density varies. As a result, these regions may differ in glass transition temperature. The use of dynamic mechanical analysis reveals these different relaxation regions occur by overlapping peaks in damping factor curves. In Figure 4, such a curve is presented. It shows an unsymmetrical peak of the damping factor in the region of glass transition. Furthermore, the peak can be separated into at least 3–4 individual peaks. As described, these individual peaks represent different fractions of the network with individual crosslink density.

### 2.5. Application of DMA Analysis on Epoxy Based Materials

Epoxy based thermosets are often used for insulating applications. One of the utilized epoxy matrix systems are epoxy-amine thermosets. The formation of a homogenous network of DGEBA based epoxy-amine systems only occurs on a stoichiometric mixture. Preparing non-stoichiometric mixtures with varying amine concentrations results in decreasing glass transition temperatures for hypo- and hyperstoichiometric amine content (see Table 1) [8,9]. Additionally, this leads to increasing network inhomogeneity (broader glass transition with more than one relaxation process). Also, blending of epoxy based thermosets with other polymeric materials may lead to network inhomogeneity [10]. However, this inhomogeneity depends on the polarity interactions between the two polymeric systems [11,12,13]. In addition to the influence of reaction stoichiometry, the incorporation of microscale or nanoscale fillers may also have an influence on the glass transition temperature of homogenous networks [14,15] as well as using different types of plasticizer [16].

Due to the fact that DGEBA is based on bisphenol A, which seems to have severe impacts on animals’ and human health [19,20], researchers have been trying to create substitutes for DGEBA based epoxy thermosets. In recent years, bio-based polymers derived from renewable resources have become increasingly important as sustainable and eco-efficient products to replace the petrochemical-derived DGEBA. Until now numerous bio-based epoxy resins deriving from vegetable oils—such as soybean oil, linseed oil, and castor oil—have been studied [17,21,22,23]. These types of epoxy resins contain linear aliphatic chains and cannot lead to glass transition temperatures above 100 °C. However, these linear systems (if not blended with other monomers) easily form homogenous networks. Using epoxy monomers based on Isosorbide leads to glass transition temperatures above 100 °C [24]. Table 1 sums up some glass transition values found in literature for various epoxy based formulations.

## 3. Mechanical Properties of Thermosets

In electric and electronic applications, the mechanical properties are a subordinate factor to the electrical performance. However, for moldings used in connectors, housings, and cases, the mechanical properties like impact properties or tensile/bending strength and elongation to failure are important. In flexible printed circuits (FPC), brittle thermoset polymers are not desirable, as these circuits are often undergoing static or dynamic loading during their lifespan. In both cases flexibility is a requirement for the resins used in the adhesive and dielectric films.

In the following sections, a brief description of the basic principles of mechanical behavior of thermosets is given, including tensile, bending, and impact behavior. Since the temperature range in thermosets’ applications lies mostly below the glass transition temperature [25], the majority of the discussions in this section will be focused on the mechanical behavior at glassy state.

### 3.1. Tensile tests on Thermosets

Among static and quasi-static testing and measuring methods, the tensile test is regarded as the most fundamental test in mechanical material testing. In contrast to the fact that pure tensile loading is rather uncommon in reality, this test ranks high in polymer testing. Various approaches to execute tensile tests are possible, requiring different specimens, loading conditions and/or clamping devices [26]. Due to the narrow elastic deformation range of plastics, the Young’s modulus is determined as a secant modulus. It includes the elastic and linear-viscoelastic deformation range of the stress–strain diagram. Evaluation is limited to the deformation range between 0.05% and 0.25% of the strain.

(1) $$E=\frac{\sigma_{2}-\sigma_{1}}{\varepsilon_{2}-\varepsilon_{1}}$$

**Figure 5:**
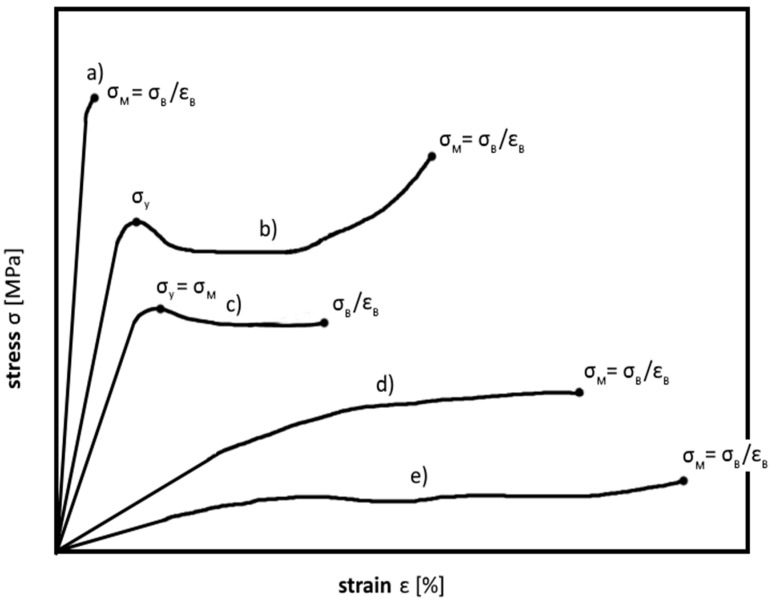
Stress–strain curves of various polymeric materials, (**a**) brittle materials; (**b**,**c**) tough materials with yield point; (**d**) tough material without yield point and (**e**) elastomeric materials.

The decrease of Young’s modulus, with increasing test temperature, can be qualitatively represented by the storage modulus in Figure 2. Because the different regions of tensile behavior are associated with different magnitudes and amounts of molecular motions and relaxation effects, it is apparent that anything that alters the molecular motions also alters the regions of tensile behavior. Thus, molecular structure, molecular weight (for thermoplastics), crosslink density (for thermosets), and test temperature can significantly influence these behaviors [5]. Tensile tests for determining strength and deformation properties of plastics are usually performed at a testing speed of 1–500 mm/min. Typical stress–strain diagrams for various plastics are shown in Figure 5. The parameters derivable from the curves correspond to characteristic points in the diagrams. Diagram (a) can be assigned to brittle material behavior with relatively high tensile strength (σ_M_), whereby the tensile strain at break (ε_B_) achieved can be as much as 10%. Typical examples for such material behavior include thermosets, as well as filled and reinforced plastics. Stress–strain diagrams of types (b) through (d) represent ductile deformation behavior with strains at break of several hundred percent, but relatively low tensile strengths. Some typical examples for such material behavior include polyolefines and polyamides. Particularly thermoplastics with type (b) and (c) stress–strain behavior exhibit a yield stress (σ_y_), at which local necking followed by a constant stress plateau occurs, also called cold yielding. The stress plateau is the result of stretching with yield zone formation, whereby the material is stretched and pulls itself simultaneously out of the unstretched part of the specimen. The type (e) stress–strain diagram in Figure 5 corresponds to the typical curve of rubbery materials with very low strength and modulus, but very high tensile strains at break. This material group includes, for example, polyvinyl chloride, as well as natural and synthetic rubber [25,26].

### 3.2. Application of Tensile Tests on Epoxy Based Materials

The effects of crosslink density or conversion on Young’s modulus, tensile strength, and elongation at break have been covered by various authors. With an increase in crosslink density or conversion, the Young’s modulus and the tensile strength are increased—whereas the elongation is decreased. This is caused by the evolution from a very soft and ductile material (at low crosslink density or conversion) to a very hard and rigid material [27]. Blending the DGEBA based epoxy materials with aliphatic or cyclic epoxy-species results in a significant change in Young’s modulus, tensile strength and elongation at break [28,29]. Through the growing hype of nanoparticles in the last centuries, various nanoparticle types like particles based on nanosilicates, carbon-nanotubes, fullerenes, etc., were incorporated into epoxy matrices to increase the material’s properties. Some of them, like carbon-nanotubes, tend to increase Young’s modulus and tensile strength at preservation of the elongation at break at filler contents below 1 w% [30]. An undoubted challenge when using layered nanoparticles is the exfoliation property of these particles, especially in the case of layered nanosilicates [31,18]. In the field of bio based DGEBA substitutes, tensile tests were also performed covering influences on tensile properties of a second reactive epoxy species, which increases Young’s modulus and tensile strength but decreases elongation [18]. Also, the influence of nanoparticles on the tensile properties of bio based epoxies was studied, revealing increases for Young’s modulus and tensile strength. However, at higher filler content the tensile strength is lower, due to the lack of particle exfoliation [32]. Table 2 illustrates values of Young’s Modulus for different types of epoxy based materials.

### 3.3. Bending Test on Thermosets

Flexural loading is one of the most common types of load encountered in electric/electronic applications. Thus it is highly significant for determining characteristic values of polymers and composite materials [25]. The quasi-static bend test is used especially for testing brittle materials, like thermosets, whose failure behavior causes technical problems with tensile tests. In actual testing practice, three-point and four-point bend test equipment is available for performing such tests. In view of the occurring loads, the four-point bend test is the fundamentally more suitable method, due to the constant bending moment and the freedom from transverse force [26]. The general differential equation of the elastic bending line, the elasticity modulus (*E*_f_), is acquired under the same condition as the tensile test [25,26]:

(2) $$E=\frac{\sigma_{f2}-\sigma_{f1}}{\varepsilon_{f2}-\varepsilon_{f1}}$$

**Figure 6:**
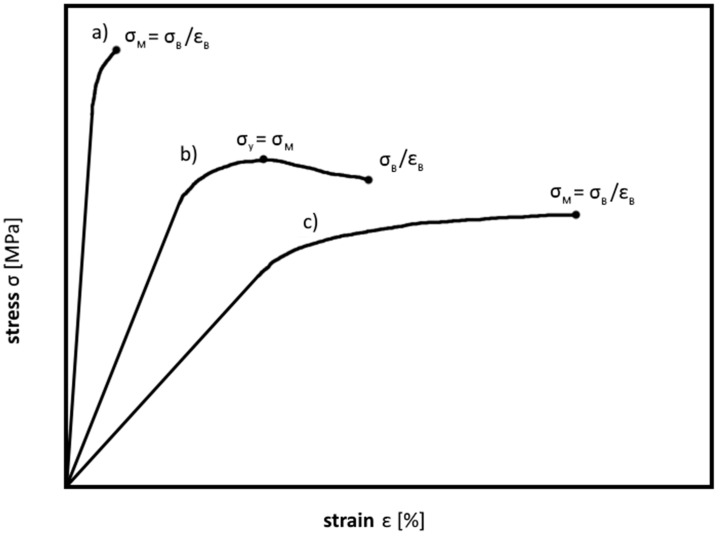
Flexural stress–strain curves of various polymeric materials, (**a**) brittle materials; (**b**,**c**) tough materials.

Figure 6 shows typical flexural stress–strain diagrams of various polymers. For the characteristic points in the diagram see the tensile test section.

### 3.4. Application of Bending Tests on Epoxy Based Materials

Compared to the tensile testing method, the same structure-property relationships also hold for bending tests. That means that an increase in crosslink density raises the flexural modulus and the bending strength—whereas the maximum flexural strain is lowered [8]. Also, blending the resin with linear reaction species results in a softening of the material (modulus and strength decreases whereas the strain is increased) [16]. Besides the use of nanoparticles as fillers to modify the resulting properties of the composite [30,31], short or endless fibers can be used to drastically change the mechanical behavior of composites. Fiber types like glass or carbon fibers drastically increase modulus and strength but lower the flexibility dramatically [34,35]. In the field of bio based materials, the use of hemp, sisal, or jute fibers are common and the resulting property changes are well described in literature [36,37]. Table 3 illustrates values of flexural modulus for different types of epoxy based materials.

### 3.5. Impact Behavior of Thermosets

**Figure 7:**
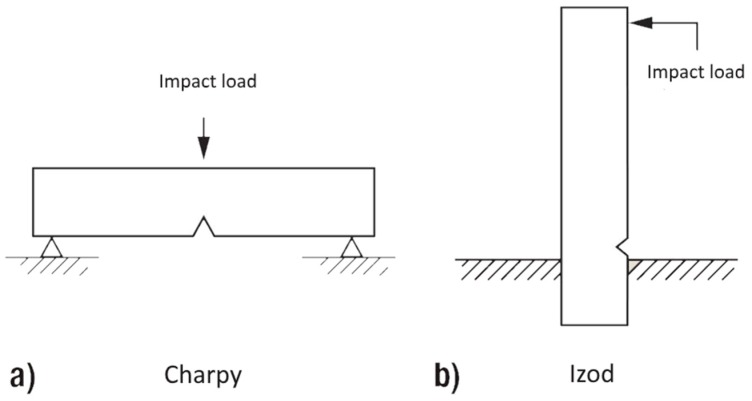
Impact loading in (**a**) Charpy and (**b**) Izod configuration.

In many applications thermosets have to withstand impact load due to environmental or application influences. Even a very small amount of impact energy could seriously reduce the static strength of materials, decreasing component reliability; any attempt to improve the tensile property would lead to a decrease of impact property at the same time. The impact toughness can be determined by measuring the needed energy to break a standard specimen, which is one of the most common methods to evaluate impact properties. Charpy (simple supported beam load; Figure 7a) and Izod (cantilever beam load; Figure 7b) impact tests were developed for isotropic materials [25]. To determine the Charpy impact strength of an unnotched specimen (*a*_cU_), the energy (*W*_c_) absorbed by breaking the specimen is related to the initial cross-section area of the specimen:

(3) $$a_{\mathrm{cU}}=\frac{W_{c}}{b*h}$$

Notched Charpy impact strength (*a*_cN_) is calculated from the absorbed energy (W_c_), related to the smallest initial cross-section of the specimen at notch base:

(4) $$a_{\mathrm{cN}}=\frac{W_{c}}{b_{N}*h}$$

The difference between Charpy impact strength *a*_cU_ and notched Charpy impact strength *a*_cN_ indicates how sensitive a material is to external notches, i.e., takes the problematic notch effect for the Charpy impact test into consideration and indicates how effective fillers are. Thus notch sensitivity can be calculated from the quotients of *a*_cN_ and *a*_cU_:

(5) $$k_{Z}=\frac{a_{\mathrm{cN}}}{a_{\mathrm{cU}}}*100\%$$

**Figure 8:**
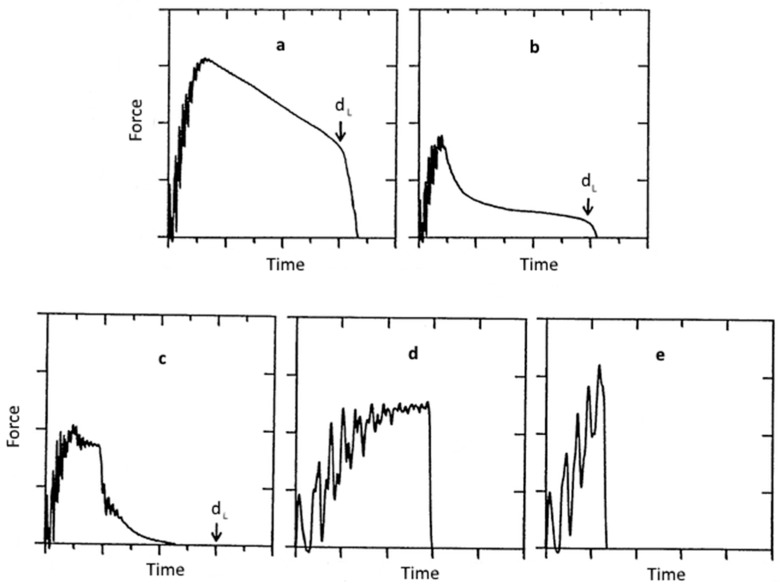
Types of failure modes in impact tests: (**a**) no break: yielding followed by plastic deformation till deflection limit d_L_; (**b**) partial break: additional yielding and stable crack growth till deflection limit d_L_; (**c**) tough break: yielding and stable crack growth till total failure; (**d**) brittle break: yielding followed by unstable crack growth; (**e**) splintering break: unstable cracking followed by yielding.

In case of inhomogeneous materials or composites (especially fire reinforced composites), fracture mode and energy absorption are affected by various test parameters, such as fiber orientation, the sample size, and the impact rate. Figure 8 illustrates the occurring failure modes in impact tests. Thermosets in the glassy state mostly feature a brittle failure mode [25,26].

### 3.6. Application of Impact Tests on Epoxy Based Materials

Basically, epoxy based materials exhibit a brittle impact behavior. However, regarding an increase in crosslink density results in a shift in brittle-to-tough transition region [24,27]. This means that with increasing crosslink density the material characteristics change from soft and ductile to brittle and rigid. Whereas a soft material can easily transform impact energy to increase molecular mobility, a rigid material needs even more energy to reach the same amount of mobility. Adding filler types like non-interactive inorganic particles to the material results in a decrease in impact property. However, by changing the interaction through surface modification or adding fibers to the material, the impact property can be highly increased [33,38,39]. In case of fibers this enhancement is caused by the crack propagation inhibition property, if they are oriented right angular to the loading direction [40]. Characterizing bio based epoxy systems the impact properties at room temperature are significantly better compared to epoxy amine systems. This is caused by their lower glass transition values and therefore higher molecular mobility [22,18]. Through the use of natural fibers like hemp, sisal, or jute fibers, the impact properties can be further increased [37]. Table 4 illustrates values of impact properties for different types of epoxy based materials.

## 4. Conclusions

This article only represents a basic overview of applicable thermo-mechanical and mechanical tests, including literature data values of epoxy amine and bio based epoxy systems for the characterization of thermoset materials. However, in the construction process of electrical applications, mechanical stresses are not considered to have severe influences on the lifetime of a product, though it is known in polymer engineering that the slightest stress or defect in polymer materials could lead to a failure of the product. Therefore, mechanical, fracture mechanical, and long-term characterization should be considered for the dimensioning of polymeric materials in electrical and electronic applications.

## Figures and Tables

**Table 1:** Glass transition values of epoxy based thermosets measured at maximum of tan δ.

Epoxy base	*T*_g_ measured at maximum tan δ (°C)	Reference
DGEBA	161	[8]
DGEBA + carbon black	169	[14]
Epoxidized allyl soyate	90	[17]
Epoxidized soybean oil	80	[18]

**Table 2:** Young’s Modulus values of epoxy based thermosets measured in tension mode.

Epoxy base	Young’s modulus (MPa)	Reference
DGEBA	2,750	[15]
DGEBA + Carbon nanotubes	3,500	[30]
DGEBA + sisal fibers	15,000	[33]
Epoxidized soybean oil	648	[18]

**Table 3:** Flexural modulus values of epoxy based thermosets measured in 3 point bending mode.

Epoxy base	Flexural modulus (Mpa)	Reference
DGEBA	3,000	[24]
DGEBA + glass fibers	4,900	[36]
DGEBA + hemp fibers	2,900	[36]
Epoxidized soybean oil	775	[18]

**Table 4:** Unnotched Charpy impact strength of epoxy based thermosets.

Epoxy base	Impact strength (kJ/m^2^)	Reference
DGEBA	11.7	[39]
DGEBA + multi walled carbnon nanotubes	23.1	[39]
DGEBA + Epoxidized soybean oil	22	[41]
